# Erythrocyte Sedimentation Rate Reference Intervals Determined via VES-MATIC 5 and CUBE 30 Touch with Respect to the Westergren Method

**DOI:** 10.3390/diagnostics15091101

**Published:** 2025-04-26

**Authors:** Maria Lorubbio, Daniela Diamanti, Carolina Pieroni, Elena Gialli, Massimiliano Pettinari, Stefania Bassi, Gabriele Gorini, Stefania Carniani, Alessandro Saracini, Paola Meloni, Michela Chiodi, Silvana Gervino, Pietro Pantone, Agostino Ognibene

**Affiliations:** 1 UOC Laboratory Medicine, Department of Laboratory Medicine and Transfusion, San Donato Hospital, 52100 Arezzo, Italy; elena.gialli@uslsudest.toscana.it (E.G.); massimiliano.pettinari@uslsudest.toscana.it (M.P.); stefania.bassi@uslsudest.toscana.it (S.B.); gabriele.gorini@uslsudest.toscana.it (G.G.); stefania.carniani@uslsudest.toscana.it (S.C.); alessandro.saracini@uslsudest.toscana.it (A.S.); paola.meloni@uslsudest.toscana.it (P.M.); michela.chiodi@uslsudest.toscana.it (M.C.); silvana.gervino@uslsudest.toscana.it (S.G.); pietro.pantone@uslsudest.toscana.it (P.P.); agostino.ognibene@uslsudest.toscana.it (A.O.); 2 Diesse-Diagnostica Senese S.p.A., 53035 Monteriggioni, Italy; danieladiamanti@diesse.it (D.D.); carolinapieroni@diesse.it (C.P.)

**Keywords:** erythrocyte sedimentation rate (ESR), EDTA tube, reference intervals, reference method, Westergren

## Abstract

**Objectives**: The erythrocyte sedimentation rate (ESR) is a diagnostic test that is employed worldwide to assess a patient’s inflammatory status. Like all laboratory tests, it requires reference intervals (RIs) to support clinical decision making and facilitate accurate diagnosis. In this study, we aimed to generate RIs for the automatic analyzers VES-MATIC 5 (VM5) and CUBE 30 touch (C30T) compared to the gold standard method. **Methods**: A total of 989 (presumably healthy) participants from Arezzo Hospital in Italy were enrolled. The ESR RIs were established according to CLSI for all three methods. **Results**: The analysis pointed out significant differences between women and men and age-related increases in ESRs obtained via all three analytical methods. The average and median values resulting from VM5 and C30T were, respectively, 1 mm/h smaller and higher than the gold standard. The RIs were calculated based on three clusters: the first pertained to patients aged ≥ 18 but ≤ 49 years; the second pertained to patients aged ≥ 50 but ≤ 69 years; the last comprised patients aged ≥ 70 years. Due to the clear overlap between these ranges and the statistical analysis, we identified only one range for females ≥ 18 years (Westergren: 1–22 mm/h; VM5: 1–22 mm/h; C30T: 1–25 mm/h). For the male participants, two separate RIs were proposed: one for those aged ≥ 18 but < 69 years (Westergren: 1–14 mm/h; VM5: 1–14 mm/h; C30T: 1–18 mm/h) and one for those aged 70 years or above (Westergren: 1–22 mm/h; VM5: 1–23 mm/h; C30T: 1–29 mm/h). **Conclusions**: The proposed RI for automated analyzers C30T and VM5 agreed with the reference method and can be adopted to measure ESRs within EDTA blood samples.

## 1. Introduction

The erythrocyte sedimentation rate (ESR) was initially adopted over a century ago, when it was first identified as an inflammation index. Westergren was the first clinician to make the association between the health state of a patient and the speed with which red blood cells (RBCs) attempt to precipitate; this finding originated in the plasmatic phase in an examination of anticoagulated blood samples from tuberculosis patients [1]. Later, Westergren proposed a technique to standardize turning these observations into a disease index.

Despite the non-specificity of the test, over the years, numerous researchers have confirmed diagnostic and prognostic applications of ESR in multiple chronic pathologies, such as rheumatological symptoms, autoimmune conditions, infections, and tumours [2,3,4]. In several consecutive guidelines published over a period of more than four decades, the International Council of Standardization in Haematology (ICSH) created an established protocol for the manual method [5,6,7], and the Westergren technique became the gold standard for measuring ESR. Unfortunately, this method proved too laborious and time-consuming, required an impractical amount of blood, and was deemed unsafe for operators, so automated ESR methods were developed to provide more objective and rapid results using the same amount of blood drawn for other diagnostic tests.

In addition to the Westergren method, the ICSH recognized two other categories of automated instrumentation for ESR determination: “modified Westergren methods” based on a modification of the sedimentation time or anticoagulants and alternate methods [6,8] based on centrifugation or photometric rheology and different anticoagulants. Moreover, the ICSH recommended assessing the analytical performance of the novel ESR analyzers with respect to the gold standard by establishing and verifying reference values obtained from healthy people. Such values should be determined locally and calculated on an independent basis for each laboratory based on the method used [8].

Two ESR analyzers have recently appeared on the market: VES-MATIC 5 and CUBE 30 touch (DIESSE Diagnostica Senese SpA, Monteriggioni, SI, Italy). These methods are considered to be modified because they are based on ESR optical readings obtained via EDTA anticoagulated sample tubes in as little as 20 min [9,10,11,12,13]. The aim of this study was to generate reference intervals (RIs) for the automatic analyzers VES-MATIC 5 (VM5) and CUBE 30 touch (C30T) compared to the gold standard method in order to support the diagnostic process in laboratory settings.

## 2. Materials and Methods

### 2.1. Participant Selection and Study Design

Outpatients and donors from the Blood Drawing and Transfusion Centres of the San Donato Hospital in Arezzo were recruited between October 2023 and May 2024. The enrolled subjects were males (*n* = 521) and females (*n* = 468) aged between 18 and 100 years, who were presumed to be healthy according to self-declaration and/or hematological tests. The selected patients had no known diseases, and pregnant women and subjects under therapy monitoring were excluded; all hematological parameters were treated as requested within the reference range. Only residual blood samples, with an initial CBC request, were used, in compliance with the Declaration of Helsinki. The study was authorized by the local ethics committee (opinion n. 23775). Bloods were collected in the morning under fasting conditions in 3.0 mL BD Vacutainer^®^ K2-EDTA tubes (Becton Dickinson, BD-Plymouth, UK) and analyzed within 2 h of venipuncture. The ESR was determined using C30T, VM5, and the Westergren method based on within-sample stability [14] and according to ICSH recommendations [8].

### 2.2. Westergren Method

The reference method was implemented according to the ICSH guidelines [5]. The blood sample was manually diluted with sodium citrate solution (at a ratio of 4:1) using the FL MEDICAL system (FL MEDICAL Torreglia, PD, Italy). This allowed the RBCs to separate from the plasma. After one hour, the distance of the plasma column from the top to the sedimented RBC layer was measured and expressed as mm/h.

### 2.3. ESR Automatic Analyzers

C30T and VM5 are ESR analyzers based on a modified version of the Westergren method, which allows the use of an EDTA blood collection tube. They determine the ESR values based on the difference between the measure of plasma column height immediately after mixing tubes and after 20 min of sedimentation using an optical reader system. The acquisitions are converted mathematically into Westergren values, and the results are expressed in mm/h. The instruments do not consume blood or produce waste, which means that they are environmentally friendly and do not pose any danger to laboratory personnel. The main differences between them are the throughput and the sample loading approach; specifically, 60 samples/h are required for C30T with manual tube insertion, whereas 190 samples/h are obtained for VM5, and the rack can be loaded directly from the CBC cell counter.

### 2.4. Study Design

Based on reports from the literature regarding the positive correlations with age and sex, all participants were divided into 6 groups of 10 years (18–29, 30–39, 40–49, 50–59, 60–69, ≥ 70) and separated by sex to identify statistically significant differences among the classes and allow us to calculate the respective RI.

Furthermore, a parallel analysis was conducted on three different age groups, similarly to other authors [7,10,15], considering an initial group of subjects aged between ≥ 18 and ≤ 49 years; a second group of subjects aged between ≥ 50 and ≤ 69 years; and finally, a group of subjects aged ≥ 70 years for females and males [16,17,18].

### 2.5. Statistical Analysis

The anonymized data were collected in a single Excel file (Microsoft Office 365), and statistical analysis of the results was conducted using MedCalc version 22.023 (MedCalc Ltd.) and SPSS V. 20.0 (IBM SpA) software.

The lower (1st, 2.5th) and upper (99th, 97.5th) reference limits and the corresponding 90% confidence intervals (CIs) were calculated in accordance with the Clinical and Laboratory Standards Institute (CLSI) guide EP28-A3C [16].

Age- and sex-specific partitions were identified and statistically confirmed using the Harris and Boyd method, and the normal distribution of each age partition was determined using the Shapiro–Wilk test. Outliers were removed using the Tukey or adjusted Tukey test. Furthermore, the Kruskal–Wallis test and Dunn’s post hoc analysis were used to calculate the significance between the different age groups in women and men in the three different analysis modalities, and Spearman’s test was used to investigate the possible correlation between ESR and hemoglobin and haematocrit.

## 3. Results

**Figure 1:**
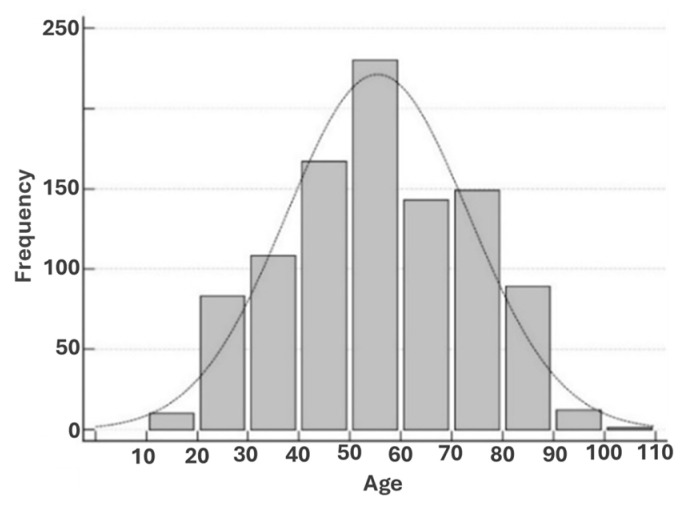
Age distribution of subjects enrolled for ESR determinations.

The ESR was measured in 989 subjects (468 females and 521 males) using three methods (C30T, VM5, and the Westergren method). The normal age distribution of the entire population is presented in Figure 1.

The means and medians were as follows: for C30T, 8 and 6 mm/h (Lower Limit, LL, 1 mm/h; Upper Limit, UL, 32 mm/h); for VM5, 6 and 4 mm/h (LL, 1 mm/h; UL, 27 mm/h); and for Westergren, 7 and 5 mm/h (LL, 1 mm/h; UL, 29 mm/h).

**Figure 2:**
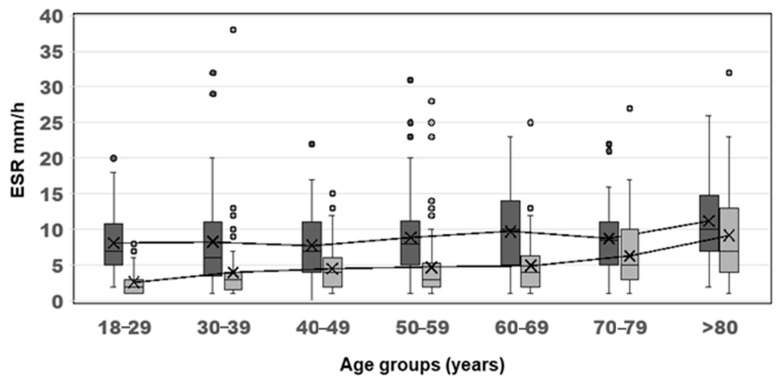
ESR media trend with age according to the Westergren method. The ESR increased with age for both women (mean 8 mm/h to 11 mm/h) and men (mean 3 mm/h to 9 mm/h). Dark grey: female. Light grey: male. The symbol X in the boxes represents the average.

**Figure 3:**
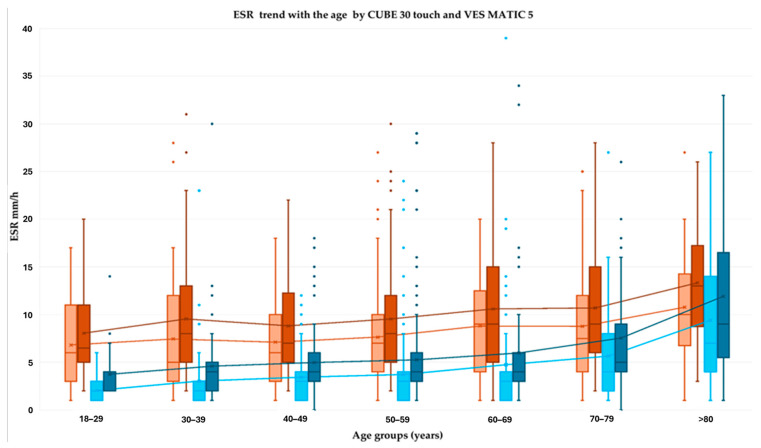
ESR media and median trend with age for each sex, measured using both automated analyzers: CUBE 30 touch and VESMATIC 5. Orange: VM5 female ESR data; red: C30T female ESR data; sky blue: VM5 male data; blue: C30T male data.

To appreciate the ESR trend with regard to age- and sex-related differences, the ESR measurements were analyzed within 10-year age bands, and the Westergren values were plotted (Figure 2) separately from the automated analyzers’ values (Figure 3).

According to the Westergren data, the ESR showed a statistically significant increase with age, and a greater increase was observed in females (median values from 8 to 11 mm/h) than in males (median from 3 to 9 mm/h). The same trends were observed for C30T data from females (median values from 8 to 13 mm/h) and males (median from 4 to 11 mm/h) as well as for VM5 data from females (median values from 7 to 11 mm/h) and males (median from 2 to 10 mm/h).

The Kruskal–Wallis test and Dunn’s post hoc analysis showed significance between the age groups of 10 years, both in women and men, for the three methods. Table 1 reports the significance between different age groups in women and the ESR values calculated at 2.5–97.5° and 1–99° using the C30T and VM5 instruments and via the Westergren method. The same data for males are presented in Table 2.

The reference ranges were calculated by dividing the population by gender. The two clusters were divided into three subgroups based on age and established according to the literature [7,10,15]. The first group included subjects aged ≥ 18 and ≤ 49 years; the second group was composed of participants aged ≥ 50 and ≤ 69 years; and the last group included subjects aged ≥ 70 years (Table 3). The lower and upper limits and CI (90%) were determined according to the CLSI guidelines [16].

Based on the significance results in the different age groups (Table 1 and Table 2) and supported by the clear overlap in the ranges that emerged during the literature-based analysis, additional reference intervals 1–99° (90% IC) were recalculated for each method and for both sexes. Only one ESR RI was suggested for women >18 years. For men, two groups were proposed. The first had an age range of 18–69 years, while the second group was composed of participants aged >70 years (Table 4).

The complete blood count was used to correlate hematocrit and hemoglobin with the ESR determined according to the methods mentioned above. The correlation with haematocrit and ESR, considering all subjects and determined with C30T, VM5, and the Westergren method, was shown to be statistically significant (r = −0.51; *p* < 0.001, r = −0.49; *p* < 0.001, and r = −0.50; *p* < 0.001, respectively). The same statistical significance was detected when the correlation was verified with hemoglobin and ESR determined via C30T, VM5, and Westergren (r = −0.51; *p* < 0.001, r = −0.50; *p* < 0.001, and r = −0.52; *p* < 0.001, respectively). Statistically significant correlations with haematocrit and hemoglobin were identified even when categorized by gender and/or age. 

Finally, the correlation was deemed statistically significant when calculated between platelets and ESR determined using the C30T, VM5, and the Westergren method, r = 0.10; *p* < 0.01, r = −0.11; *p* < 0.015, and r = 0.14; *p* < 0.003, respectively. The slight significance was not confirmed when considering gender.

## 4. Discussion

In this study, we defined the ESR RIs for the C30T and VM5 methods and the Westergren method. The C30T and VM5 methods are automated, whereas the Westergren method is performed manually. These analyzers, according to a multicenter study [13], have been successfully used for precision analysis, conducted for 5 days with high and low ESR controls, intra-run and inter-run, with CVs consistently lower than 10% for both VM5 and C30T. However, higher CVs were observed at very low ESR levels, where small variations in results can lead to higher standard deviations in line with other studies [10,11,13]. Repeatability for both instruments was also observed with CVs < 10%. Regarding the accuracy assessment, a Spearman coefficient (R2) of 0.978 and 0.981 was observed between VM5 and C30T compared to the reference methods, respectively.

To avoid bias in the determination of RIs, considering that the study population was composed solely of blood donors, we included both donors and random outpatients [19]. To represent the reference population, reference samples were selected a priori and a posteriori [20].

The mean and median values for the whole study group were evaluated to assess the potential differences in automated analyzers compared to the manual method. Data obtained with VM5 and C30T were 1 mm/h lower and 1 mm/h higher, respectively, than the results of the gold standard (7 and 5 mm/h, respectively), confirming that their performance was satisfactory [13].

Based on previous scientific observations present in the literature, gender and age differences were also considered. All three methods showed that the ESR differences between women and men remained consistent throughout all age ranges (Figure 2 and Figure 3). Reference intervals were calculated for each method and for both sexes, considering three age-based subgroups which are frequently studied in the literature: the first group included subjects aged 18 to 49; the second group comprised people aged between 50 and 69; and the third group was made up of people aged 70 and over (Table 3).

The three female RIs computed for the C30T were about 2 mm/h higher than those obtained for the Westergren method in the upper limits, while the male RIs showed a difference of 9 mm/h only in patients over the age of 70. The VM5 RIs were smaller than the gold standard for patients of both sexes who were younger than 69 years of age. In the elderly, the two methods exhibited remarkably similar results.

In compliance with the statistical significance derived from the different age groups (Table 1 and Table 2) and the observation of the excessive overlap between the RIs classes calculated via literature-defined clusters, a unique RI for women and RIs for men from two age classes were proposed (respectively, ≥18 years and ≥ 18 ≤ 69; ≥70) as shown in Table 4.

In healthy subjects, the ESR differences between women and men, as well as the respective age-related increases, are largely ascribed to the direct correlation with increased fibrinogen levels and the inverse relationship with changing hemoglobin levels, which fluctuate over the course of a person’s life [15,21,22,23]. Additionally, the increasing trend of the ESR could be the result of the physiological changes in plasma protein, serum markers, and hormone levels during normal ageing processes, which also involve blood rheology [24,25,26]. Moreover, gender hormonal differences influenced ESR: women tend to have a higher baseline than men [15,22,24,27].

Years later, our Westergren data agreed with previous research [15], and only minimal differences, which lacked clinical relevance, were reported for the median and upper limits. The RIs proposed in 1967 were 0–15 mm/h (mean 5 mm/h) for the 20–49 age group and 0–22 mm/h (mean 7 mm/h) for the 50–69 age group; for women, the suggested ranges were 0–24 mm/h (mean 9 mm/h) for participants aged 15–49 years and 0–32 mm/h (mean 12 mm/h) for those aged 50–69 years. Individuals aged 70 years or older were not considered. Regarding the ESR in the elderly, the Westergren data were below the mean and upper limits with respect to values shown in other works that utilized the same technique [28].

The RIs proposed in the last century were likely obtained using a non-standardized variation in the Westergren technique and with materials and consumables that differ from those used today, as opposed to our study, which was performed in agreement with ICSH guidelines [5,6] and focused on populations living in other geographic locations. An investigation such as ours, which was conducted on the Norwegian population, noted the same slight decrease with respect to another study performed in 1965 on the same population [29,30], and their data were similar to ours. Both discrepancies obtained with the most recent studies could be related to changes in lifestyle and eating habits, so it is important to demonstrate that a better living condition is associated with a reduction in ESR [21].

Our confirmation of manually obtained RIs is a useful verification, because nowadays, most of the reference limits adopted in laboratory settings are selected from the literature data [3,15,29].

Although numerous researchers have utilized C30T in recent years, none of them have examined the RIs of this analyzer [11,12,13]. Our study is the first to validate the RIs obtained using the automated analyzer: for female participants ≥ 18 years, we obtained a value of 1–25 mm/h. For males, two separate RIs were obtained: 1–18 mm/h in subjects ≥ 18 but >69 years and 1–29 mm/h in participants aged 70 years or older.

In addition, a recent VM5 validation work verified [10] the upper reference limits for the instrument. The results presented in the cited study were significantly higher than the values presented in Table 3 and Table 4. Differences were recorded for all age groups in both women and men, using the VM5 method and the Westergren method, and in the mean and upper limit. The bias was probably due to the smaller and not sufficiently differentiated population enrolled for RI verification. The study also reported that VM5’s ESR values were higher than Westergren’s; this contradicts our result.

In addition, our results were obtained using a modified Westergren method. They were significantly different from the IRs obtained by alternate methods [18], which proposed RIs for men of 2–28 mm/h (15–50 years) and 2–39 mm/h for the 51–70 age group, while for women, the range was 2–37 mm/h for those aged 15–50 and 2–39 mm/h for the 51–70 age group. In addition, individuals of both sexes who were over the age of 70 were considered as a single group with only one RI (3–46 mm/h), while our investigation suggested separate RIs for the older sex groups with age differences. Moreover, the overestimation observed with the alternate method with respect to the manual and modified methods had already been reported in the same sample cohort [9]. This evidence supported the ICSH recommendation to validate or verify the RIs for a new ESR analyzer in order to achieve the best diagnostic interpretation.

Our study confirms that ESR undergoes systemic variation throughout a person’s lifetime, but to our knowledge, it does not affect ultradian and circadian rhythms in a significant way. The absence of these effects and the increases in values that occur throughout the life course are likely intrinsic to the nature of ESR as a physical phenomenon related to the plasma proteins and to factors that control RBC aggregation. Additional investigations should be conducted to explore ESR tuning during the day and its possible relationship with dietary factors. For all of the proposed reference ranges, there were limitations, and an adequate ESR baseline value for everyone could likely be determined by serial measurements made over time, as suggested by Coskun [31] in their study on the necessity of providing personalized RIs using individual data [19,32,33,34].

This study has several limitations. Firstly, it primarily focuses on individuals from a specific region (Arezzo, Italy), which could limit the generalizability of the results to larger populations with different demographics. Though our statistics may help to define the validity and significance of the representativeness of the studied sample, the population considered in this study will not be representative of the entire planet; as such, each laboratory is required, according to the recommended guidelines, to establish its own ranges [16]. Another limitation is that this is a cross-sectional study, which does not account for changes in ESR readings over time in the same individuals, which could provide insight into physiological changes. Therefore, future studies with a longitudinal design could be performed to observe how ESR values fluctuate over time within individuals, especially in those with known inflammatory conditions.

The validation of reference intervals in different geographical and demographic contexts requires more research to understand how ESR values may vary across different populations and assess the impact of factors such as diet, lifestyle. In addition, future longitudinal studies will examine ESR dynamics over time in same individuals and include various health statuses to provide a more holistic view.

## 5. Conclusions

The definition of RIs is the starting point for assessing a physiological condition or supporting a therapeutic decision, including ESRs, although the test is a measure of a biological phenomenon rather than a direct measurement of an analyte. The proposed RIs for the C30T and VM5 automated analyzers were in agreement with those obtained using the manual reference method and can be adopted by laboratories that measure ESRs on blood samples of EDTA using these modified methods. In the meantime, our data could also support laboratories that are still using the Westergren method.

## Figures and Tables

**Table 1:** Significance by age range (18–29, 30–39, 40–49, 50–59, 60–69, >70) in women, determined via the Kruskal–Wallis test, with a significance level *p* < 0.0001 and post hoc analysis (Dunn) for C30T and VM5 instruments and the Westergren method. ESR values calculated at 2.5–97.5° and 1–99° were also reported. ≠: different.

Factor Age Groups (Years)	*N*	≠ From Factor nr	Centiles of ESR_C30T_F				Centiles of ESR_VM5_F				≠ From Factor nr	Centiles of ESR_ Westergren_F			
			***0.01***	***0.025***	***0.975***	***0.99***	***0.01***	***0.025***	***0.975***	***0.99***		***0.01***	***0.025***	***0.975***	***0.99***
**1 (18–29)**	54	(7)	1	1	18	19	1	1	17	19	(7)	1	1	17	19
**2 (30–39)**	47	(7)	1	1	19	21	1	1	17	19	(7)	1	1	17	19
**3 (40–49)**	70	(7)	1	1	20	22	1	1	18	20	(7)	1	1	18	20
**4 (50–59)**	112	(7)	1	1	21	23	1	1	18	20	-	1	1	18	20
**5 (60–69)**	61	-	1	1	22	24	1	1	19	21	-	1	1	19	21
**6 (70–79)**	70	-	1	1	24	26	1	1	20	23	-	1	1	20	23
**7 (>80)**	54	(1)(2)(3) (4)	1	1	26	28	1	1	22	24	(1)(2)(3)	1	1	21	23

**Table 2:** Significance for age range (18–29, 30–39, 40–49, 50–59, 60–69, >70) in males calculated with Kruskal–Wallis test, with a significance level *p* < 0.0001 and post hoc analysis (Dunn) for C30T and VM5 instruments and the Westergren method; ESR values calculated at 2.5–97.5° and 1–99° were also reported. ≠: different.

Factor Age Groups (Years)	*N*	≠ From Factor nr	Centiles of ESR_C30T_M				Centiles of ESR_VM5_M				≠ From Factor nr	Centiles of ESR_Westergren_M			
			*0.01*	*0.025*	*0.975*	*0.99*	*0.01*	*0.025*	*0.975*	*0.99*		*0.01*	*0.025*	*0.975*	*0.99*
**1 (18–29)**	39	(6)(7)	1	1	8	8	1	1	5	5	(3)(4)(5) (6)(7)	1	1	6	7
**2 (30–39)**	61	(6)(7)	1	1	10	11	1	1	7	8	(6)(7)	1	1	10	11
**3 (40–49)**	97	(6)(7)	1	1	11	12	1	1	9	10	(1)(7)	1	1	11	13
**4 (50–59)**	118	(6)(7)	1	1	12	13	1	1	11	12	(1)(6)(7)	1	1	11	13
**5 (60–69)**	82	(7)	1	1	14	15	1	1	13	14	(1)(7)	1	1	12	13
**6 (70–79)**	77	(1)(2)(3) (4)	1	1	19	21	1	1	17	19	(1)(2)(4)	1	1	15	17
**7 (>80)**	47	(1)(2)(3) (4)(5)	1	1	28	31	1	1	22	25	(1)(2)(3) (4)(5)	1	1	22	25

**Table 3:** Reference values proposed at 1–99° [IC 90%] and calculated using the specified age groups in females and males based on the literature. RIs were obtained according to CLSI C28-A3 [16].

	*N*	RIs ESR (mm/h) C30T		RIs ESR (mm/h) VM5		RIs ESR (mm/h) Westergren	
Centiles		0.01 [IC 90%]	0.99 [IC 90%]	0.01 [IC 90%]	0.99 [IC 90%]	0.01 [IC 90%]	0.99 [IC 90%]
**Female**							
**Aged ≥ 18 ≤ 49 years**	171	1 [1–1]	23 [22–25]	1 [1–1]	20 [20–22]	1 [1–1]	21 [19–22]
**Aged ≥ 50 ≤ 69 years**	173	1 [1–1]	25 [24–27]	1 [1–1]	22 [21–23]	1 [1–1]	21 [20–23]
**Aged ≥ 70 years**	124	1 [1–1]	26 [25–29]	1 [1–1]	23 [21–24]	1 [1–1]	22 [21–25]
**Male**							
**Aged ≥ 18 ≤ 49 years**	197	1 [1–1]	13 [11–15]	1 [1–1]	10 [8–12]	1 [1–1]	13 [10–17]
**Aged ≥ 50 ≤ 69 years**	200	1 [1–1]	17 [15–20]	1 [1–1]	17 [13–21]	1 [1–1]	16 [13–19]
**Aged ≥ 70 years**	124	1 [1–1]	29 [23–33]	1 [1–1]	23 [19–27]	1 [1–1]	22 [18–25]

**Table 4:** Reference values proposed at 1–99° [IC 90%] calculated by age and categorized in male participants (two groups) and female participants (one group). The reduction in RI classes in both sexes is suggested by the overlap between distributions at 90% shown in Table 3. Reference values were obtained according to the CLSI standard C28-A3, which revealed the statistical significance between groups (see Table 3 and Table 4).

	*N*	RIs ESR (mm/h) C30T		RIs ESR (mm/h) VM5		RIs ESR (mm/h) Westergren	
Centiles		0.01 [IC 90%]	0.99 [IC 90%]	0.01 [IC 90%]	0.99 [IC 90%]	0.01 [IC 90%]	0.99 [IC 90%]
**Female**							
**Aged > 18 years**	468	1 [1–1]	25 [23–26]	1 [1–1]	22 [21–23]	1 [1–1]	22 [21–23]
**Male**							
**Aged >1 8 < 69 years**	397	1 [1–1]	18 [14–21]	1 [1–1]	14 [12–17]	1 [1–1]	14 [12–17]
**Aged ≥ 70 years**	124	1 [1–1]	29 [23–33]	1 [1–1]	23 [19–27]	1 [1–1]	22 [18–25]

## Data Availability

The dataset used during this study is available from the corresponding authors upon reasonable request.
